# Evaluation of a Novel Method for Temporary Eyebrow Lifting Using Polydioxanone Threads: Preliminary Findings

**DOI:** 10.3390/jcm14020490

**Published:** 2025-01-14

**Authors:** Kyu Hwa Jung, Won Lee, Seong Hwan Kim

**Affiliations:** 1 Liting Plastic Surgery Clinic, Seoul 06035, Republic of Korea; millgaloo@naver.com; 2 Yonsei E1 Plastic Surgery Clinic, Anyang 14072, Republic of Korea; e1clinic@daum.net; 3 Kangnam Sacred Heart Hospital, Hallym University College of Medicine, Seoul 07441, Republic of Korea

**Keywords:** aging, thread lifting, eyebrow shape, polydioxanone thread, patient satisfaction

## Abstract

**Background/Objectives:** Aging affects the face and eyebrow areas, with various resultant procedures for lifting the eyebrows. Recently, thread lifting using absorbable threads has become increasingly popular, with the advantages of a faster recovery and no visible scars, when compared with conventional facial rhytidectomy. Furthermore, polydioxanone (PDO) thread lifting is a favorable surgical method that has been used for eyebrow lifting. However, simply raising the eyebrows overall does not always result in high patient satisfaction. Therefore, in this study, we successfully applied a surgical method to lift the eyebrows, achieving a softer impression of the changing eyebrow shape that is associated with aging. We report on the favorable results yielded by the application of this surgical method. **Methods:** Between January 2023 and January 2024, a retrospective chart review was conducted for 29 patients who had undergone eyebrow lifting using only PDO threads. Photographs were taken pre- and 3 months post-operatively, in a photo studio with indirect lighting. Patient satisfaction and adverse effects were evaluated immediately and 3 months post-operatively. **Results:** The overall change in the eyebrow height was minimal at approximately 1.2–1.3 cm; nonetheless, the changes in the angles of the inner eyebrows at 4.00° and 4.44° resulted in a more favorable appearance. In total, 26 out of 29 patients expressed being “satisfied” or “very satisfied” with the outcomes. Serious complications were not observed. **Conclusions:** An effective, noninvasive eyebrow correction, considering the shape of the inner eyebrow, was performed. A thread-lifting method was used, which lifted the overall eyebrows and corrected the eyebrow shape.

## 1. Introduction

Facelift surgery is one of the most commonly performed procedures in cosmetic surgery [1]. Thread lifting using absorbable threads has become increasingly popular in recent years, with the advantages of a faster recovery and no visible scars, when compared with conventional facial rhytidectomies [2]. Moreover, polydioxanone (PDO) thread lifting has been used for eyebrow lifting [3]. This is due to the periorbital area being one of the most noticeable parts of an aging face, impacting first impressions [4]. Age-related changes in the eyebrow shape can create an expression of sadness or hostility. Thus, correcting this area is essential [5]. Eyebrow ptosis is a well-known, age-related condition. Endoscopy-assisted forehead rejuvenation with an eyebrow lift has widely been utilized; however, limitations thereof include a relatively long recovery time and high costs [6]. As an alternative, noninvasive techniques using energy-based devices and botulinum toxin injection have been introduced; nonetheless, limitations exist regarding the duration of the effects thereof [7,8]. Furthermore, methods using hyaluronic acid fillers or absorbable threads have been implemented to achieve sustained and immediate effects [9,10].

However, regardless of the method used to achieve successful eyebrow lifting, complaints largely arise regarding an excessively surprised look or unnatural eyebrows that do not appear youthful. This is postulated to be due to a singular focus on lifting to correct eyebrow ptosis, while neglecting age-related changes in the eyebrow shape. Several studies have discussed that, with age, the lateral aspect of the eyebrows tends to undergo relative lowering [11,12,13].

**Figure 1:**
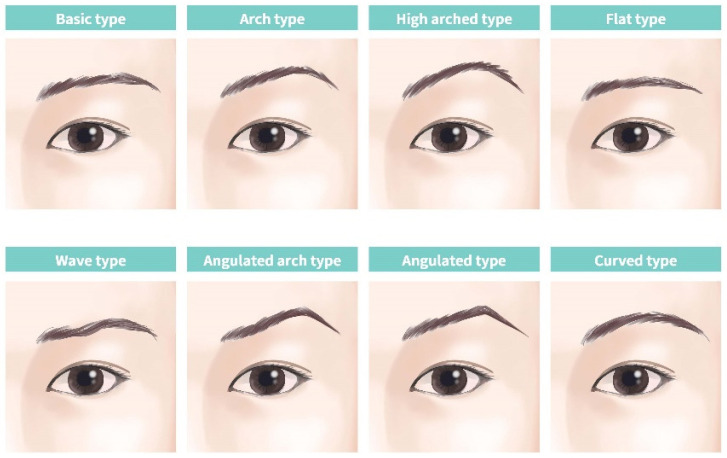
Classification of the eyebrow shapes.

Nevertheless, in clinical practice, many patients consulting at a hospital have eyebrows with a common shape where the medial aspect is low and the peak point is raised, commonly referred to as “seagull eyebrows”. This eyebrow shape gives an angry or unattractive impression to Asians. Eyebrows can be classified into various types; however, eyebrows categorized as angulated or high-arched convey a strong impression (Figure 1). Thus, we considered how simply modifying the eyebrow angle can change the appearance of the eyebrow and result in a younger appearance and increased patient satisfaction, regardless of whether the eyebrow is substantially elevated or not.

Therefore, in this study, we successfully applied a surgical method to lift the eyebrows, achieving a softer impression of the changing eyebrow shape that is associated with aging. We report on the favorable results yielded by the application of this surgical method.

## 2. Materials and Methods

### 2.1. Ethical Considerations

This study was approved by the Institutional Review Board of the Hallym National University Hospital Ethics Committee (No. 2024-02-029). All procedures involving human participants were performed in accordance with the ethical standards established by the institutional and/or national research committee, and with the 1964 Helsinki Declaration and its later amendments or comparable ethical standards.

### 2.2. Study Design

Between January 2023 and January 2024, a retrospective chart review was conducted for 29 patients who had undergone eyebrow lifting using only PDO threads. The inclusion criteria were as follows: patients with blepharochalasis were included, while those with blepharoptosis were excluded. Patients with a history of forehead surgery or those who had received fillers in the forehead were also excluded. However, patients who had undergone botulinum toxin treatments were also included. Additionally, male patients with completely fixed deep forehead wrinkles and patients with excessively thin skin, where the threads might be visible, were excluded from the study.

The materials used included a double-arm bidirectional cog thread (21-gauge, 300 mm in length, USP 1, Sthepharm, Seoul, Republic of Korea) and six bidirectional cog threads (19-gauge, 70 mm in length, USP 2-0, Sthepharm, Seoul, Republic of Korea).

**Figure 2:**
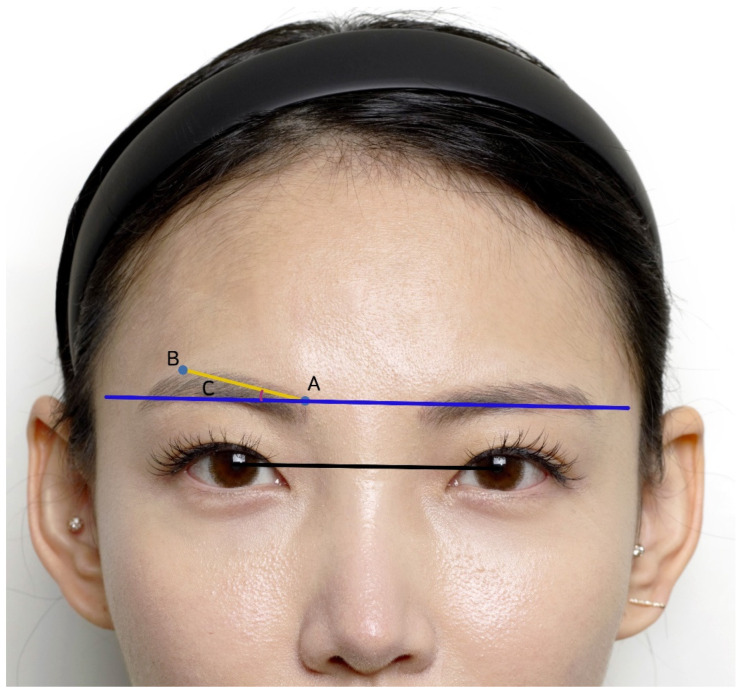
Schematic diagram of the reference points. The black line represents the mid-pupillary axis; the blue line represents a virtual horizontal line from the inner eyebrow area; point A represents the medial end of the eyebrow; point B represents the highest point of the eyebrow; and C corresponds to the angle between the virtual horizontal line and the line connecting points A and B.

Photographs were taken pre- and 3 months post-operatively, in a photo studio with indirect lighting (Canon EOS 800D Camera, EF-S18-55 mm, F3.5-5.6 IS II). The evaluation of the photographic results was based on setting the mid-pupillary axis as the horizontal reference line (Figure 2, black line) and establishing a virtual horizontal line from the inner eyebrow area (Figure 2, blue line).

Furthermore, a virtual line connecting the upper medial margin of the eyebrow (Figure 2, indicated by A) to the highest point of the eyebrow (Figure 2, indicated by B) was drawn, and the angle with the horizontal line was measured (Figure 2, indicated by C). The changes in the angle and eyebrow height from the mid-pupil were objectively measured, pre- and 3 months post-operatively. Finally, patient satisfaction was evaluated immediately and 3 months post-operatively. A 4-point questionnaire was administered, as follows: 3 = “very satisfied”, 2 = “satisfied”, 1 = “barely satisfied or dissatisfied”, and 0 = “worse”.

### 2.3. Surgical Technique

**Figure 3:**
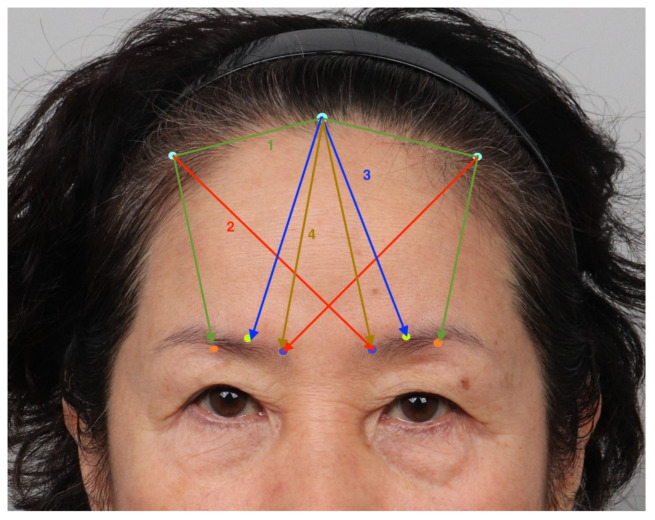
Schematic diagram of the surgical procedure. The first step involves performing an overall eyebrow lifting using a double-arm long thread (green arrow, 1), with the sequential insertion of multidirectional cog threads (2, 3, and 4).

Three entry points were created along the hairlines. The first entry point was marked at the center, and two additional entry points were marked slightly lateral to the superior temporal septum, on the hairline. The patient was requested to frown to induce contraction of the frown muscles, allowing identification of the position of the transverse and oblique heads of the corrugator supercilii muscle. A puncture was made at the entry point using an 18-gauge needle, with subsequent widening using Metzembaum scissors. A double, long-threaded needle was inserted through the central entry point and removed through the lateral puncture site. The needle was reinserted to emerge from the lower border of the eyebrow at the midpoint. This thread lifted the eyebrow overall and was finally trimmed to an upright position (Figure 3, green arrow).

Subsequently, three bidirectional cog threads (19-gauge, 70 mm in length, USP 2-0) were used to elevate the medial aspect of the eyebrow. The first thread was inserted from the outer hairline entry point to the medial aspect of the opposite eyebrow and was trimmed (Figure 3, orange arrow). The second 70 mm length thread was inserted from the central entry point of the hairline, removed at the transverse head of the corrugator supercilii muscle, and trimmed (Figure 3, blue arrow). Finally, the thread was inserted from the central entry point to the medial end of the eyebrow (Figure 3, dark green arrow). The overall surgical procedures are included in Supplemental Video S1.

### 2.4. Statistical Analysis

A paired *t*-test was used to determine if the therapy resulted in improvements. The results were analyzed using IBM SPSS Statistics for Windows (version 22.0; IBM, Armonk, NY, USA). Statistical significance was defined as *p* < 0.05.

## 3. Results

**Figure 4:**
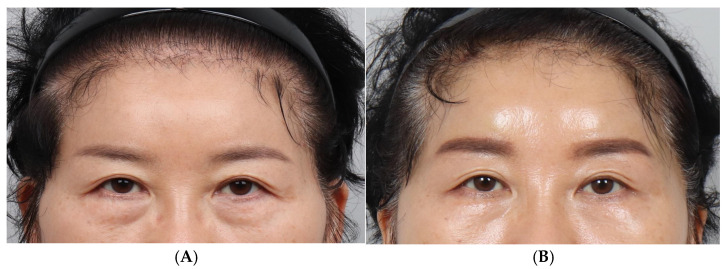
Photographs corresponding to a 61-year-old female patient who underwent forehead thread lifting. (**A**) In the pre-operative view, the angle of the right eyebrow is 1° and that of the left eyebrow is 12°. (**B**) Three months post-operatively, the eyebrow angle has changed to 9° on the right and 10° on the left eyebrow. The eyebrow height has increased from 1.2 cm to 1.5 cm, revealing an elevation of 0.3 mm. The amount of elevation is very small; nonetheless, an overall change in the eyebrow shape is observed. Thus, the patient looks younger, with a resultant high level of satisfaction.

**Figure 5:**
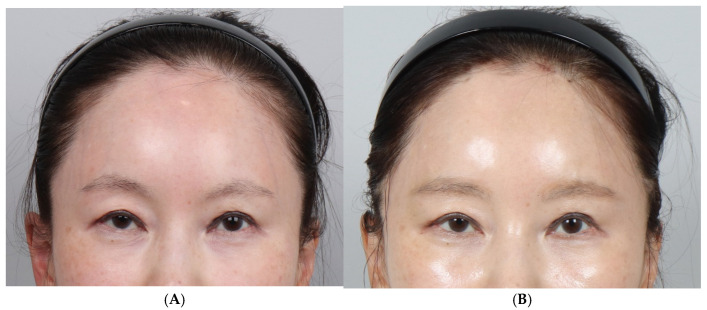
A 53-year-old female patient. (**A**) Pre-operative right angle: 27°; left angle: 25°. (**B**) Post-operative 3 months right angle: 19°; left angle: 15°.

A total of 29 female patients underwent cogged thread eyebrow lifting between January 2023 and January 2024 (Figure 4 and Figure 5). Their ages ranged from 58 to 84 years old, with an average age of 67.6 years old.

The angle of the right eyebrow pre-operatively was 16.10° and was post-operatively reduced by 4.00° to 12.10°, while the angle of the left eyebrow decreased from 15.5° to 11.06°, a reduction of 4.44° (Table 1). Both changes were statistically significant at the 95% confidence interval. These results indirectly indicated that patient satisfaction was influenced by changes in the angle of the medial eyebrow. Moreover, improving the inward tilt of the eyebrow, due to aging, was as important as simply lifting the eyebrows. Changes in the eyebrow height were observed, with an increase of 0.12 cm in the right eyebrow from 1.42 cm to 1.55 cm, and an increase of 0.13 cm in the left eyebrow from 1.43 cm to 1.56 cm (Table 2). These changes were statistically significant at the 95% confidence interval.

**Figure 6:**
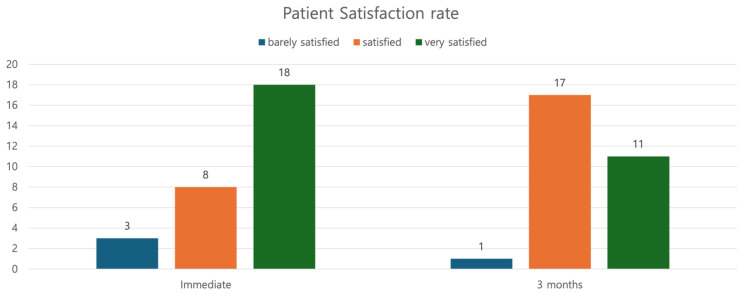
Satisfaction scores.

The satisfaction rate immediately after the procedure averaged at 2.52. At 3 months post-operatively, the satisfaction rate decreased to 2.35, nevertheless indicating a relatively high level of satisfaction. Among the twenty-nine patients, three indicated a satisfaction score of 1, or “barely satisfied”, immediately post-operatively (Figure 6). Conversely, regarding their satisfaction with the outcomes of the procedure, the remaining 26 patients responded with “satisfied” or “very satisfied”. Of the three patients who responded with “barely satisfied” immediately post-operatively, one patient developed a skin dimple, and irregularities were observed in the other two patients (Table 3). The skin dimple that had been observed in the one patient appeared at the site where the thread crossed the glabellar area. This was postulated to have occurred as the cannula passed superficially through that area and was easily resolved with a gentle massage.

The skin irregularities observed in the remaining two patients were sufficiently mild that the patients did not notice them unless under certain lighting conditions and naturally resolved over time. Furthermore, no major adverse effects, such as hematoma, nerve injury, severe swelling, or persistent pain, were observed.

## 4. Discussion

As the aging process progresses, various facial changes occur at the individual and racial levels. While wrinkles commonly cause a person to appear older, the overall change in facial shape has a greater impact on aging [14]. Both the facial shape and eyebrows tend to droop, due to the overall forehead and skin laxity [15]. Eyebrow correction has mainly been performed through lifting procedures; nevertheless, cases have occurred with unsatisfactory clinical results. Authors have attributed this to the lack of consideration regarding changes in the eyebrow shape. Consequently, in our study, forehead thread lifting was performed, taking into consideration the shape of the eyebrows, which resulted in favorable outcomes and high patient satisfaction.

Changes in the eyebrow shape due to aging result from tissue sagging, exhibiting different patterns of change depending on the medial, central, and lateral parts of the eyebrow, as described in several studies [16,17,18]. The shape of the eyebrow is influenced by facial bones and soft tissue, in addition to the essential balance between the upward force of the frontalis muscle and downward force of the glabellar frown muscles. The shape of the inner aspect of the eyebrow is determined by the relatively weak upward pull of the frontalis muscle and downward forces exerted by the corrugator and depressor supercilii and procerus muscles, in addition to the medial bands of the orbicularis oculi muscle (OOM). Additionally, the middle to lateral thirds are determined solely by the force of the frontalis muscle, while the outer part of the eyebrow is relatively less influenced by this muscle [19].

Some studies have described that eyebrow height increases with age [20], and the shape and height of the eyebrows are influenced by the imbalance of muscle forces. Most prevalent is the spastic frontalis syndrome, in which the chronic activation of the frontalis muscle to overcome the weakness of the levator system results in an elevated eyebrow height and a more arched brow appearance. Additionally, increased activity of the corrugator muscle and OOM causes the inner aspect of the eyebrow to descend, while the frontalis muscle pulls the eyebrow upward, from the center to the outer aspect. As this phenomenon continues, the shape of the eyebrow becomes fixed [21]. Such eyebrow deformities convey negative expressions, such as angry or sad appearances. Therefore, older patients prefer flat eyebrows [22].

The most widely used noninvasive method for correcting age-related eyebrow shape is the botulinum toxin injection; however, a short duration of effect exists. Occasionally, the botulinum toxin disrupts the balance of muscles, resulting in unanticipated expressions. Methods such as the use of energy-based devices and thread lifting have been introduced; nonetheless, most studies have mainly focused on lifting the eyebrows. Surgical methods are more effective and long-lasting; however, recovery time, complications, cost, and scarring must be considered.

In the present study, the method implemented by the authors has the advantage of correcting the eyebrow shape while performing eyebrow lifting and is considered particularly effective for patients with age-related eyebrow drooping. Furthermore, unlike conventional forehead thread lifting, this method creates a crisscross point, providing a longer duration for the lifting effect [23].

Nevertheless, the effectiveness thereof may be limited in cases of severe soft tissue laxity or when the horizontal wrinkles on the forehead are already static. Additionally, irregularities from thread lifting may occur in patients with very thin skin. Some patients experienced slight pain when the forehead muscles moved; however, most healed naturally with no serious adverse effects.

Threads designed for lifting purposes can be classified based on their raw materials, which can be broadly categorized into polydioxanone (PDO), Poly-L-Lactic Acid (PLLA), Polycaprolactone (PCL), and Polypropylene (PP). Among these, the most commonly encountered thread in the South Korean lifting market is PDO threads. PDO is a polymer composed of repeating ether–ester units, obtained through the ring-opening polymerization of the monomer p-dioxanone. PDO typically exhibits about 55% crystallinity and has the advantage of relatively high strength, although it may vary slightly depending on the manufacturing process. Additionally, it is known for its excellent biocompatibility and low toxicity. The melting point of PDO is reported to be around 110 degrees Celsius, which is lower than that of PGA and PLLA but higher than that of PCL. However, one limitation of PDO is its degradation period of 4 to 6 months, with strength retention lasting approximately 1.5 months. After this period, about 60% of its strength remains, gradually decreasing thereafter. When discussing the duration of effects, it is important to consider the perspective from which the evaluation is made. The duration of immediate effects can be maintained without change for up to 2 months, but after that, they gradually diminish, with a slow loss occurring between 6 months and 1 year. However, while the physical effects may fade, the benefits of neocollagenesis in preventing tissue loosening may extend to 1.5 years. If non-absorbable threads are used, the physical effects may last longer, but they are also expected to diminish between 1.5 and 2 years.

Therefore, when performing thread lifting with the goal of achieving more long-term effects, it is reasonable to explain and conduct the procedure with consideration for the chemical effects of neocollagenesis rather than focusing solely on the immediate effects. Repeated thread-lifting procedures can accumulate tightening and lifting effects, leading to longer-lasting results.

This study has several limitations that warrant consideration. Firstly, the sample size was limited to 29 patients, which may not provide a comprehensive assessment of the interventions’ effectiveness across a broader population. Additionally, the follow-up period was inadequate; exceeding one year is essential to ensure a thorough evaluation of potential complications and to address uncertainties regarding the long-term benefits of repeat procedures. Therefore, further studies with larger patient populations and longer follow-up periods are necessary to achieve a more complete understanding of the safety and efficacy of the proposed methods. The effectiveness of thread lifting may also vary according to the patient’s age, underscoring the importance of further investigations in this regard.

## 5. Conclusions

As aging progresses, changes occur in the upper face, and the shape of the eyebrows changes, due to the imbalance of the muscles. The authors performed an effective, noninvasive eyebrow correction, considering the shape of the inner eyebrow. A thread-lifting method was used, which lifted the overall eyebrows and corrected the eyebrow shape.

## Figures and Tables

**Table 1:** Pre- and post-operative results of the medial eyebrow angle differences are presented.

	Pre-Operative (*n* = 29)	Post-Operative (*n* = 29)	Difference (SE∆ **)	*p*-Value
Right eyebrow angle *	16.10 ± 6.00	12.10 ± 5.03	4.00 (0.54)	<0.05
Left eyebrow angle *	15.51 ± 5.59	11.06 ± 4.45	4.44 (0.53)	<0.05

* Data are reported as means ± standard errors. The data have been compared using paired *t*-tests. ** Standard error with a change or difference.

**Table 2:** Pre- and post-operative differences in eyebrow height.

	Pre-Operative (*n* = 29)	Post-Operative (*n* = 29)	Difference (SE∆ **)	*p*-Value
Right eyebrow height *	1.42 ± 0.34	1.55 ± 0.30	0.12 (0.04)	<0.05
Left eyebrow height *	1.43 ± 0.34	1.56 ± 0.32	0.13 (0.04)	<0.05

* Data are reported as means ± standard errors. The data have been compared using paired *t*-tests. ** Standard error with a change or difference.

**Table 3:** Complications.

Type of Complication	Number of Events
Skin dimple	1
Irregularity	2
Hematoma	0
Severe swelling	0
Nerve injury	0
Persistent pain	0

## Data Availability

The data presented in this study are available on request from the corresponding author.

## Supplementary Materials

The following supporting information can be downloaded at https://www.mdpi.com/article/10.3390/jcm14020490/s1, Video S1: The video of overall surgical procedure.
